# Case Report: Single-port robotic-assisted partial splenectomy for a giant congenital splenic cyst in a child using the SHURUI system

**DOI:** 10.3389/fped.2026.1836401

**Published:** 2026-06-12

**Authors:** Qingchi Zhang, Lingling Sun, Zhongxi Zhang, Kuiran Dong

**Affiliations:** 1 Department of Pediatric Surgery, Children’s Hospital of Fudan University (Xiamen Branch), Xiamen Children’s Hospital, Xiamen, China; 2 Xiamen Key Laboratory of Pediatric General Surgery Diseases, Xiamen, China; 3 Department of Pediatric Surgery, Children's Hospital of Fudan University, Shanghai, China

**Keywords:** case report, child, congenital splenic cyst, partial splenectomy, SHURUI system, single-port robotic surgery

## Abstract

**Introduction:**

Congenital splenic cysts are rare in children. When cysts become large or symptomatic, surgical intervention may be required. Because total splenectomy increases the risk of overwhelming post-splenectomy infection, spleen-preserving procedures are generally preferred whenever technically feasible. With the development of minimally invasive surgery, robotic platforms may provide technical advantages in complex spleen-preserving procedures. However, experience with single-port robotic surgery for pediatric splenic cysts remains extremely limited.

**Case description:**

We report the case of a 9-year-old boy weighing 33.5 kg who presented with progressive abdominal distension. Preoperative ultrasonography and computed tomography demonstrated a giant splenic cystic lesion with marked mass effect on adjacent structures. Given the lesion size and the need to preserve splenic function, single-port robotic-assisted partial splenectomy using the SHURUI system was performed. The cyst was decompressed first, followed by selective control of the segmental splenic vessels with hemoclips and demarcation-guided parenchymal transection. The operation was completed successfully without conversion or intraoperative complications. Estimated blood loss was approximately 20 mL, and the patient was discharged on postoperative day 6. Pathological examination, supported by immunohistochemistry, confirmed a congenital epithelial splenic cyst. At 3-month follow-up, the patient remained asymptomatic, with satisfactory residual splenic morphology and no evidence of recurrence. Additionally, he had resumed competitive sports without any limitations at 6-month follow-up.

**Discussion:**

This case suggests that single-port robotic-assisted partial splenectomy using the SHURUI system is feasible in selected pediatric patients with giant congenital splenic cysts. The procedure provided adequate exposure, precise dissection, and satisfactory spleen preservation through a transumbilical approach. Although the experience is limited to a single case and longer follow-up is required, this report adds to the emerging evidence supporting robotic spleen-preserving surgery in children.

## Introduction

Congenital splenic cysts are uncommon in children and are usually detected incidentally or after progressive enlargement causes abdominal distension, pain, or compression symptoms (1–3). These lesions include true cysts with an epithelial lining and pseudocysts without such lining (4–6). Management depends on cyst size, symptoms, location, and the amount of splenic parenchyma that can be preserved (2).

Conventional laparoscopic approaches have improved postoperative recovery and cosmetic outcomes, but partial splenectomy for large cysts remains technically demanding, particularly when the lesion is close to the splenic hilum or involves the upper pole (7–15). In recent years, spleen-preserving surgery has become increasingly favored for benign pediatric splenic lesions because preservation of splenic tissue helps maintain immunological function (12, 15).

Robotic systems may facilitate these procedures by providing improved dexterity, enhanced visualization, and more precise dissection (16–19). However, reports of pediatric single-port robotic-assisted partial splenectomy remain scarce. Here, we describe a child with a giant congenital epithelial splenic cyst who underwent successful single-port robotic-assisted partial splenectomy using the SHURUI system. The unique features of this case include the pediatric age, the giant cystic lesion with diagnostic challenges on preoperative imaging, the need for spleen preservation, the pathological confirmation with immunohistochemical support, and the use of a novel single-port robotic platform. This report highlights the technical feasibility and short-term favorable outcome of this approach in a carefully selected child.

## Case description

### Patient information

A 9-year-old boy weighing 33.5 kg was admitted with progressive abdominal distension. There was no history of abdominal trauma, fever, parasitic disease, or previous abdominal surgery. No relevant family history was identified.

### Clinical findings

Physical examination revealed fullness in the left upper abdomen without signs of peritonitis. No obvious tenderness, rebound tenderness, or palpable superficial lymphadenopathy was noted. His general condition was stable.

### Timeline

**Figure 1:**
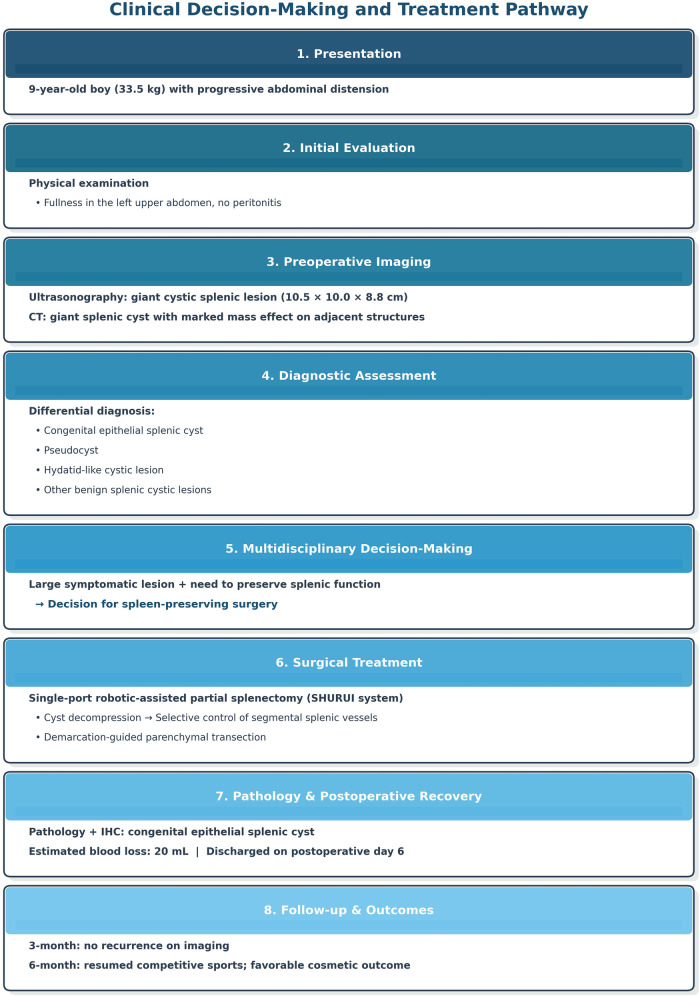
Flowchart of the clinical decision-making and treatment pathway. The patient presented with progressive abdominal distension. Preoperative ultrasonography and computed tomography demonstrated a giant splenic cystic lesion. After multidisciplinary evaluation, spleen-preserving surgery was planned. Single-port robotic-assisted partial splenectomy using the SHURUI system was performed successfully. Pathological examination with immunohistochemical support confirmed a congenital epithelial splenic cyst. The postoperative course was uneventful, and follow-up showed no recurrence and satisfactory functional recovery.

The overall clinical course is summarized in Table 1, while the key decision-making and treatment steps are illustrated in Figure 1.

**Table 1:** Timeline of the clinical course.

Time	Clinical course
Several weeks before admission	Progressive abdominal distension was noted by the family, without fever, trauma, or previous abdominal surgery.
At admission	Physical examination revealed fullness of the left upper abdomen without signs of peritonitis.
Preoperative assessment	Abdominal ultrasonography demonstrated a 10.5 × 10.0 × 8.8 cm cystic lesion, and computed tomography confirmed a giant splenic cyst involving a substantial portion of the spleen with marked mass effect on adjacent structures.
Multidisciplinary evaluation	Given the large size of the lesion and the need to preserve splenic function, spleen-preserving surgery was planned after multidisciplinary discussion and guardian consent.
Operation day	Single-port robotic-assisted partial splenectomy was performed using the SHURUI system. The cyst was decompressed first, followed by selective control of the segmental splenic vessels and parenchymal transection along the demarcation line.
Postoperative course	The operation was completed without conversion or major intraoperative complications. Operative time was 305 min, and estimated blood loss was 20 mL.
Postoperative day 6	The patient recovered uneventfully and was discharged.
3-month follow-up	The patient remained asymptomatic. Imaging showed satisfactory residual splenic morphology without evidence of cyst recurrence.
6-month follow-up	The patient had resumed normal daily activities and competitive sports without limitation, and won the U11 football championship, indicating excellent functional recovery.

### Diagnostic assessment

Preoperative abdominal ultrasonography was routinely performed and demonstrated splenomegaly with a giant cystic lesion measuring approximately 10.5 × 10.0 × 8.8 cm. The lesion had a well-defined margin, a relatively thin wall, poor internal acoustic transmission, flocculent hypoechoic components, and punctate hyperechoic foci. No obvious internal blood-flow signal was detected within the lesion, whereas slight blood-flow signals were observed along the cyst wall. The splenic hilar vessels were displaced along the outer and superior aspect of the mass, and the pancreatic tail and left kidney appeared compressed. Ultrasonography suggested a giant cystic splenic lesion, with a hydatid-like splenic cyst considered in the differential diagnosis (6).

**Figure 2:**
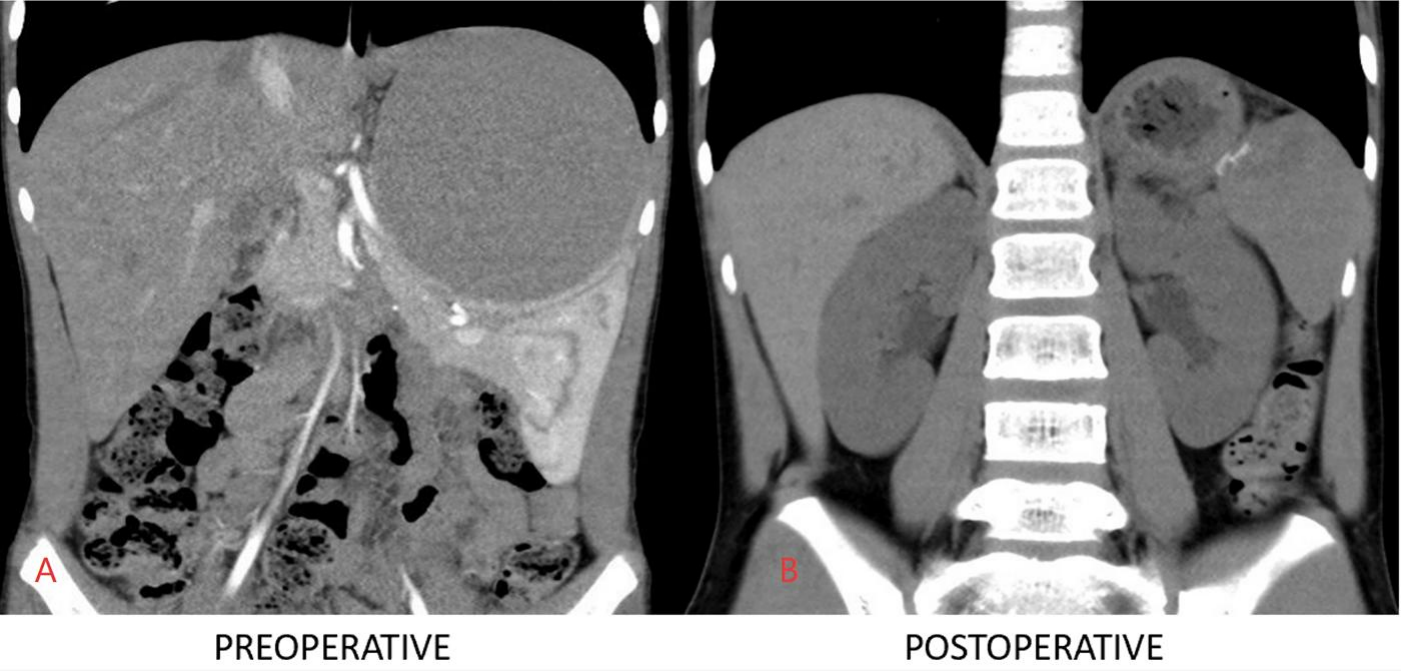
Preoperative and postoperative computed tomography findings. **(A)** Preoperative coronal computed tomography image showing a giant splenic cystic lesion with marked mass effect on adjacent structures. **(B)** Postoperative coronal computed tomography image demonstrating satisfactory residual splenic morphology without evidence of cyst recurrence.

Abdominal computed tomography further demonstrated a large cystic lesion involving a substantial portion of the spleen, with marked mass effect on adjacent structures (Figure 2A). The lesion showed imaging features suggestive of a benign splenic cyst, without obvious solid enhancing components. Based on ultrasonography and CT, the differential diagnostic considerations included congenital epithelial splenic cyst, pseudocyst, hydatid-like cystic lesion, and other benign cystic lesions arising from the spleen or adjacent structures (20, 21). Given the lesion size, mass effect, and the importance of preserving splenic function in a child, surgical treatment with a spleen-preserving strategy was recommended.

### Therapeutic intervention

After multidisciplinary evaluation and informed consent from the patient's guardians, single-port robotic-assisted partial splenectomy was performed using the SHURUI system. Ceftriaxone was administered 30 min before surgery. The SHURUI system provided high-definition endoscopic imaging with a resolution of 1,920 × 1,080, and an 85° endoscope was used. A transumbilical incision was made for placement of the single-port access device. After robotic docking, intra-abdominal exploration confirmed a giant cystic splenic lesion.

**Figure 3:**
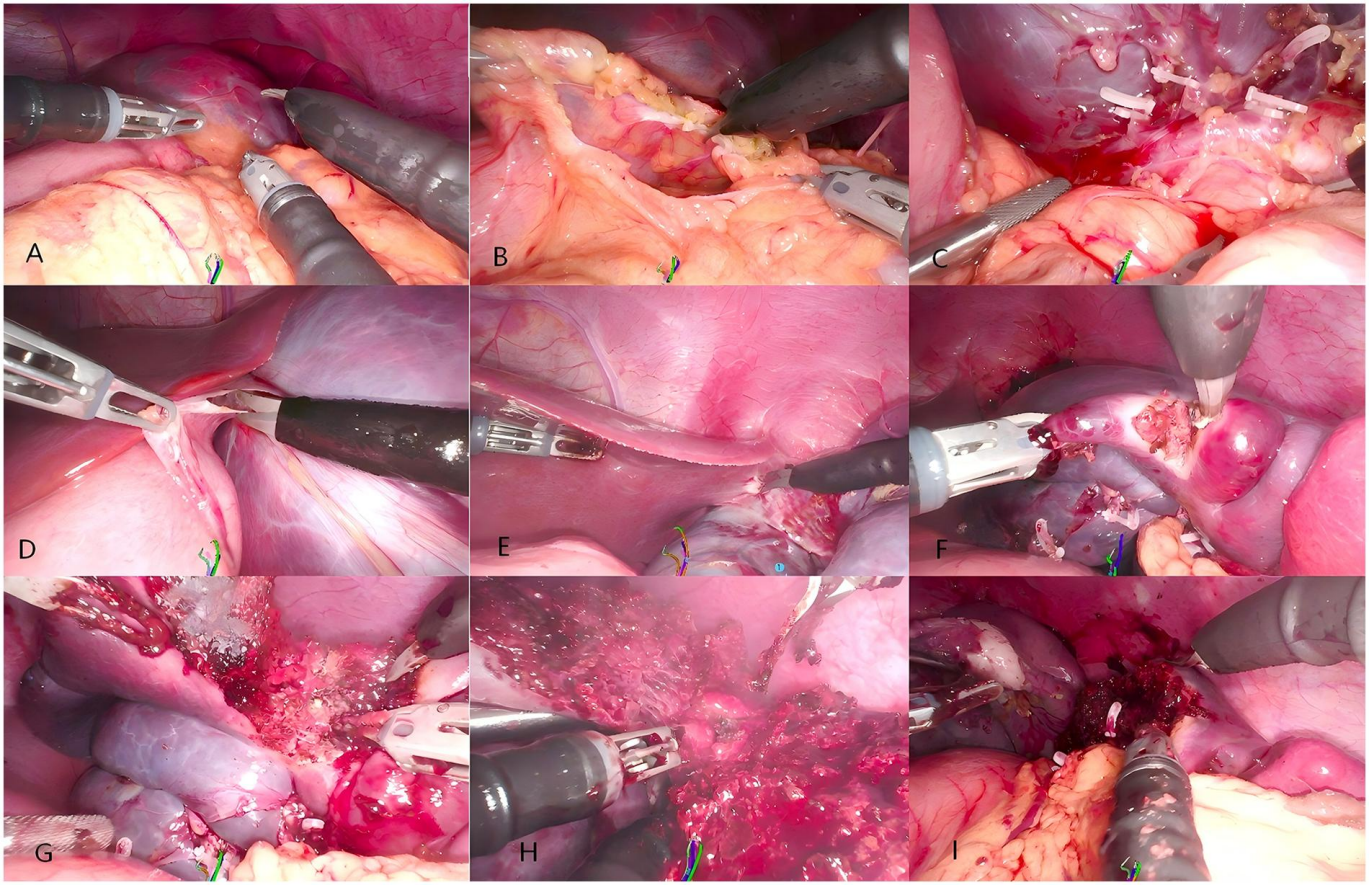
Key operative steps of single-port robotic-assisted partial splenectomy for a giant upper-pole splenic cyst. **(A)** Initial exploration revealing a giant cystic lesion located at the upper pole of the spleen. **(B)** Opening of the gastrocolic ligament to access the lesser sac and expose the splenic hilar area. **(C)** Careful dissection and control of the hilar branches supplying the involved splenic pole. **(D)** Division of the gastrosplenic ligament to further mobilize the spleen. **(E)** Division of the splenophrenic ligament. **(F–H)** Parenchymal transection along the ischemic demarcation line after selective vascular control. **(I)** Final appearance of the preserved splenic remnant.

The cyst was punctured first, and approximately 500 mL of pale yellow fluid was aspirated to reduce its volume and improve exposure. The gastrosplenic ligament was carefully dissected to expose the splenic hilum. Using articulated robotic instruments, the branches of the splenic artery and vein supplying the affected splenic pole were meticulously identified and controlled with hemoclips. Following vascular control, an ischemic demarcation line became visible on the splenic surface, which guided the resection plane. Partial splenectomy was then completed along the demarcation line with preservation of the remaining viable splenic tissue (Figure 3).

Hemostasis of the transection surface was achieved using bipolar coagulation and additional suturing when required. The splenic remnant showed satisfactory perfusion. The specimen was retrieved through the umbilical incision in a sterile endoscopic bag. A drain was placed in the splenic fossa. The operation was completed without conversion or major intraoperative complications. Estimated blood loss was approximately 20 mL, primarily based on the blood-containing volume collected in the suction canister after excluding the aspirated cyst fluid and irrigation fluid, with intraoperative visual cross-checking.

### Follow-up and outcomes

Postoperatively, ceftriaxone was continued for 3 days and then discontinued because the patient remained afebrile and showed no evidence of infection. The postoperative course was uneventful. Vital signs remained stable, oral intake was resumed gradually, and the patient became ambulatory during the early postoperative period. No fever, wound infection, intra-abdominal hemorrhage, or other adverse events occurred. The patient was discharged on postoperative day 6.

Gross examination showed splenic tissue measuring approximately 11.0 × 7.0 × 4.0 cm and weighing about 150 g. On sectioning, a large cystic lesion measuring approximately 10.0 × 6.2 × 2.2 cm was identified. The inner cyst wall was smooth, and the surrounding splenic tissue was preserved with a relatively clear boundary. Microscopically, the cyst wall was lined by stratified squamous epithelium, with fibrosis and focal hyalinization in the cyst wall and surrounding tissue. Focal hemorrhage and calcification were present within the cyst wall. Adjacent splenic tissue showed congestion, and white pulp boundaries remained identifiable. Immunohistochemical staining showed positivity for CK and CK5/6 in the epithelial lining, while Calretinin and WT-1 were negative, supporting an epithelial rather than mesothelial origin. Ki-67 showed only limited proliferative activity. These findings supported the diagnosis of a congenital epithelial splenic cyst.

**Figure 4:**
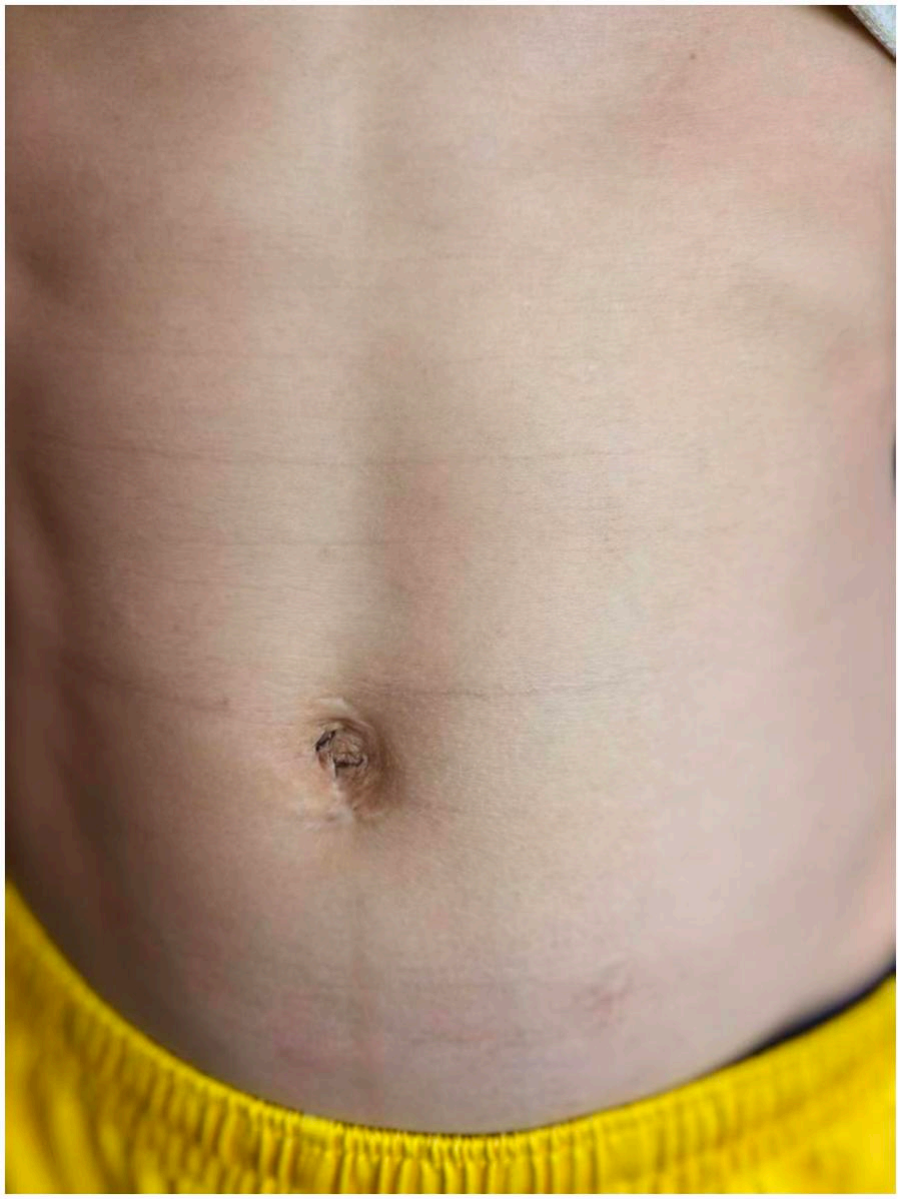
Postoperative appearance of the transumbilical incision at 6-month follow-up. The incision showed a favorable cosmetic outcome after single-port robotic surgery.

At 3-month follow-up, the patient remained asymptomatic, and imaging demonstrated satisfactory residual splenic morphology without evidence of cyst recurrence (Figure 2B). At 6-month follow-up, the patient had resumed normal daily activities and competitive sports without limitation. Notably, he returned to football and won the U11 championship. The cosmetic appearance of the transumbilical incision was satisfactory (Figure 4).

## Discussion

Congenital splenic cysts are rare in the pediatric population, and optimal management should balance definitive treatment with preservation of splenic function (1–3, 12). Observation may be reasonable for small and asymptomatic lesions, whereas larger cysts are more likely to cause symptoms, rupture, infection, hemorrhage, or progressive compression of adjacent organs and therefore often require surgical intervention (1, 2, 7, 10, 13, 14).

Historically, total splenectomy was frequently performed for large splenic cysts. However, growing recognition of the immunological importance of the spleen has shifted surgical practice toward spleen-preserving procedures whenever technically feasible (2, 12). Partial splenectomy offers the advantage of definitive lesion removal while maintaining residual splenic tissue and function, which is particularly important in children (2, 12).

Despite these advantages, partial splenectomy remains technically demanding in cases involving giant cysts, distorted anatomy, and limited operative space (7, 9, 11, 12, 15). In the present case, the lesion occupied a large portion of the upper to middle splenic pole, making exposure and vascular control challenging. Initial cyst decompression improved visualization and created working space, allowing subsequent dissection of segmental vessels and parenchymal transection along the ischemic demarcation line.

The robotic platform may be particularly helpful in this setting. The SHURUI single-port system is a purpose-built single-port robotic platform with flexible instruments introduced through a single access channel and surgeon-controlled high-definition endoscopic visualization. Compared with conventional laparoscopy, it may improve instrument articulation, camera stability, and precision during segmental vascular dissection. Compared with mature multiport robotic systems, its transumbilical single-port configuration may reduce the number of abdominal incisions, although the working space is narrower and docking, instrument collision avoidance, and team familiarity remain important technical considerations. Existing reports on the SHURUI platform in children and adults suggest that this system is technically feasible across selected single-port procedures, although pediatric experience remains limited (16–19).

Recent pediatric reports by Kong et al. described transumbilical single-site laparoscopic management of a primary splenic cyst and a splenic papillary angioendothelioma, respectively, demonstrating that single-site laparoscopic splenic surgery can provide favorable recovery and cosmetic outcomes in carefully selected children (22, 23). Compared with those laparoscopic single-site approaches, the robotic single-port approach used in the present case may offer improved dexterity and more stable visualization during selective segmental vessel control and parenchymal transection, but it also requires specialized equipment, additional docking steps, and longer procedure-specific training. Although direct comparative evidence in pediatric splenic surgery remains limited, broader pediatric minimally invasive literature suggests that both robotic and laparoscopic approaches may reduce surgical trauma in selected children, while emphasizing strict patient selection, surgeon experience, and the need for longer follow-up (24). Thus, the present case should be interpreted as a feasibility observation rather than evidence of superiority over conventional laparoscopy, single-site laparoscopy, or multiport robotic surgery.

The strengths of this report include detailed perioperative description, objective postoperative imaging follow-up, cosmetic assessment of the transumbilical incision, and pathological confirmation with immunohistochemical support. The limitations include the single-case design, lack of direct comparison with laparoscopic or multiport robotic approaches, relatively long operative time, limited follow-up duration, and the absence of formal postoperative immune-function testing. The main lesson from this case is that careful decompression, selective segmental vascular control, and demarcation-guided transection may enable successful robotic spleen-preserving management of selected giant splenic cysts in children. Further accumulation of cases and longer follow-up will be necessary to clarify reproducibility, long-term safety, cost-effectiveness, and appropriate indications.

## Patient perspective

According to the patient's guardians, the postoperative recovery was smooth, and they were satisfied with the preservation of the spleen and the cosmetic appearance of the transumbilical incision. They also reported that the child returned to normal school life and sports without limitation. At 6 months after surgery, he resumed playing football and won the U11 championship.

## Data Availability

The datasets presented in this article are not readily available because they contain de-identified clinical information from a single pediatric patient. To protect patient privacy and confidentiality, the data are not publicly available. Access may be granted by the corresponding author on reasonable request, subject to institutional approval and applicable ethical requirements. Requests to access the datasets should be directed to Kuiran Dong, kuirand@fudan.edu.cn.

## References

[1] Barakat MA Abdul-Hafez HA Sayed ASI Fahed IM Alawneh M Qarmash BA. A rare case of giant epithelial splenic cyst causing abdominal pain and anemia in a 10-year-old girl. Ann Med Surg (Lond). (2025) 87:3027–31. 10.1097/MS9.0000000000003238 40337385 PMC12055054

[2] Krichen I Maazoun K Kitar M Khiari A Sahnoun L Mekki M. Huge non-parasitic mesothelial splenic cyst in a child: a case report and literature review. Clin Med Insights Pediatr. (2021) 15:11795565211021597. 10.1177/11795565211021597 34158804 PMC8182210

[3] Febrianti M Purnomo E Dwihantoro A Makhmudi A Kashogi G. Uncommon splenic cysts in paediatric patients: a case series. Med J Malaysia. (2024) 79(Suppl 4):91–4. 39215423

[4] Abu Sabha MR Thalji M Abu Laila K Alhashlamoun M Abu Rumaila A Bannoura S. A giant non-traumatic splenic pseudocyst successfully treated with cyst aspiration and partial cystectomy: a case report and review of literature. Cureus. (2024) 16:e61110. 10.7759/cureus.61110 38919238 PMC11197975

[5] Gray TT Patel V Timpone M Felux K. Primary splenic epidermoid cyst: a case report. Cureus. (2022) 14:e22799. 10.7759/cureus.22799 35382206 PMC8976451

[6] Kabaalioğlu A Uzer E Keven A Gündüz N Doğan H Özmen E. Splenic cysts: clues in sonographic differential diagnosis and a new role for twinkling artifact. Med Ultrason. (2024) 26:125–30. 10.11152/mu-4353 38805624

[7] Tiutiuca RC Nastase Puscasu AI Stoenescu N Moscalu M Bradea C Eva I. Laparoscopic approach to primary splenic cyst: case report and review of the literature. Life (Basel). (2024) 14:120. 10.3390/life14010120 38255735 PMC10817520

[8] Vasilescu AM Târcoveanu E Ciuntu B Fotea V Georgescu S Ludusanu A. Laparoscopic approach for nonparasitic splenic cysts and splenic abscesses. Ann Ital Chir. (2022) 93:671–9. 36259435

[9] Ushakov KV Askerov RF Chundokova MA Zalikhin DV Dondup OM. Laparoscopic partial resection of spleen in a 15-year-old girl. Khirurgiia (Mosk). (2023) 7:100–5. 10.17116/hirurgia2023071100 37379412

[10] Karbasian F Ataollahi M Mashhadiagha A Moosavi SA Forooghi M Ansary N. Giant non-parasitic splenic cyst: a case report. J Med Case Rep. (2023) 17:501. 10.1186/s13256-023-04246-9 38049884 PMC10696752

[11] Sun Y Yu XF Yao H Chai C. Laparoscopic partial splenectomy for a giant splenic pseudocyst with elevated CA19-9: a case report. Ann Med Surg (Lond). (2024) 86:4849–53. 10.1097/MS9.0000000000002327 39118735 PMC11305767

[12] Wang Z Peng C Wu D Xu J Sun J Pang W. Surgical treatment of benign splenic lesions in pediatric patients: a case series of 30 cases from a single center. BMC Surg. (2022) 22:295. 10.1186/s12893-022-01745-2 35906560 PMC9335990

[13] Termos S Othman F Aljewaied A Alkhalil AM Alhunaidi M Parayil SM. Symptomatic giant primary nonparasitic splenic cyst treated with laparoscopic decapsulation: a case report and literature review. Am J Case Rep. (2020) 21:e927893. 10.12659/AJCR.927893 33211675 PMC7684427

[14] Ahmed Z Alblowi T. Laparoscopic resection of a large symptomatic splenic cyst: a case report. Cureus. (2024) 16:e54580. 10.7759/cureus.54580 38523991 PMC10957793

[15] Chaouch MA Hadj Taieb A Ben Jabra S Noomen M Zayeti M Mili E. A case report of a large splenic epidermoid cyst treated with partial splenectomy. Ann Med Surg (Lond). (2024) 86:1220–3. 10.1097/MS9.0000000000001675 38333297 PMC10849445

[16] Liang J Bai X Qin S Wen Z. The SHURUI single-port robotic system for choledochal cyst excision and biliary reconstruction in children: a case report (with video). Int J Surg Case Rep. (2024) 121:110037. 10.1016/j.ijscr.2024.110037 39013245 PMC11304057

[17] Zhang C Wang Z Jing T Wei Y Guo F Xiao C. Robot-assisted single-port retroperitoneal partial nephrectomy with a novel purpose-built single-port robotic system with deformable surgical instruments. World J Urol. (2024) 42:134. 10.1007/s00345-024-04827-3 38478100 PMC10937792

[18] Wang Z Zhang C Jing T Wei Y Xiao C Fang Y. A novel single-port robotic system in urology: a prospective multicenter single-arm clinical trial evaluating feasibility and efficacy of first 50 cases. Asian J Urol. (2025) 12:152–61. 10.1016/j.ajur.2024.07.002 40458570 PMC12126927

[19] Hu X Ruan M Zhu L Huang M Qi L Huang M. First-in-human trial and prospective case series of a novel single-port robotic system for gynaecological surgery: an IDEAL stage 2a study. Int J Med Robot. (2024) 20:e2657. 10.1002/rcs.2657 39303291

[20] Soo WT Lau KS Yong SG Soon KC. Giant epithelial nonparasitic splenic cyst: a pre-operative diagnosis dilemma. Med J Malaysia. (2021) 76:597–9. 34305129

[21] Alshammari S Alshenaifi S Alfawaz F Alkanhal A Alsaif F. A 16-year-old Saudi boy with a symptomatic large splenic epidermoid cyst. Am J Case Rep. (2021) 22:e934503. 10.12659/AJCR.934503 34759259 PMC8594136

[22] Kong M Chen S Bai Y Zhai Q Zhang S Wu Y. Transumbilical single-site laparoscopic treatment of primary splenic cyst in child: a rare case report and review of literature. Front Pediatr. (2024) 12:1454487. 10.3389/fped.2024.1454487 39386018 PMC11461222

[23] Kong M Zhai Q Zhang S Bai Y Chen S Wu Y. Transumbilical single-site laparoscopic resection of splenic papillary angioendothelioma in a pediatric patient: a rare case report and review of literature. Front Oncol. (2025) 15:1571209. 10.3389/fonc.2025.1571209 40727476 PMC12301355

[24] Merajikhah A Bastami M Mahdood B Soleimani M Ranjbar Moghaddam M Gazestani AM. A systematic review of minimally invasive laparoscopic and robotic surgery in wilms tumor. Indian J Surg. (2025) 87:992–1001. 10.1007/s12262-025-04286-z

